# L-Arginine supplementation as mitochondrial therapy in diabetic cardiomyopathy

**DOI:** 10.1186/s12933-024-02490-x

**Published:** 2024-12-20

**Authors:** Antonella Fiordelisi, Federica Andrea Cerasuolo, Roberta Avvisato, Antonietta Buonaiuto, Marianna Maisto, Antonio Bianco, Valeria D’Argenio, Pasquale Mone, Cinzia Perrino, Stefania D’Apice, Roberta Paolillo, Antonio Pezone, Fahimeh Varzideh, Gaetano Santulli, Daniela Sorriento, Guido Iaccarino, Jessica Gambardella

**Affiliations:** 1 Department of Molecular Medicine and Medical Biotechnologies, Federico II University, Naples, Italy; 2 Department of Statistics, Computer Science, Applications (DiSIA), University of Florence, Florence, Italy; 3 Department of Clinical Medicine and Surgery, Federico II University, Naples, Italy; 4 Department of Advanced Biomedical Sciences, Federico II University, Naples, Italy; 5 Federico II University Hospital, Naples, Italy; 6 Department of Public Health, Federico II University, Naples, Italy; 7 Department of Human Sciences and Quality of Life Promotion, San Raffaele Open University, Rome, Italy; 8 CEINGE- Advanced Biotechnologies, Naples, Italy; 9 Department of Medicine and Health Sciences “Vincenzo Tiberio”, University of Molise, Campobasso, Italy; 10 Casa di Cura Privata Montevergine, Mercogliano, Italy; 11 Department of Medicine (Division of Cardiology), Wilf Family Cardiovascular Research Institute, Einstein Institute for Aging Research, Albert Einstein College of Medicine, New York City, NY USA; 12 Department of Biology, Federico II University, Naples, Italy; 13 Department of Molecular Pharmacology, Einstein-Mount Sinai Diabetes Research Center (ES-DRC), Einstein Institute for Neuroimmunology and Inflammation (INI), Fleischer Institute for Diabetes and Metabolism (FIDAM), Albert Einstein College of Medicine, New York City, NY USA; 14 International Translational Research and Medical Education (ITME) Consortium, Academic Research Unit, Naples, Italy; 15 Interdepartmental Center of Research on Hypertension and Related Conditions (CIRIAPA), Federico II University, Naples, Italy

## Abstract

In patients with type II diabetes, the development of diabetic cardiomyopathy (DC) is associated with a high risk of mortality. Left ventricular hypertrophy, diastolic dysfunction, and exercise intolerance are the first signs of DC. The underlying mechanisms are not fully elucidated, and there is an urgent need for specific biomarkers and molecular targets for early diagnosis and treatment. Mitochondrial alterations play a key role in the development of DC, and microRNAs regulating mitochondrial function are emerging as potential biomarkers of metabolic stress in DC. L-Arginine (Arg) supplementation has been shown to be an effective strategy for improving mitochondrial function and energetics, with a significant impact on physical performance. The aim of the current study was to evaluate the effects of Arg supplementation on cardiac mitochondrial function, DC development, and relative phenotypes including exercise intolerance. We used db/db mice as a model of type II diabetes, chronically treated with Arg (1 mg/kg/day) for 12 weeks. Arg-treated db/db mice showed preserved diastolic function and left ventricular morphology compared with untreated diabetic mice. Arg supplementation also improved exercise tolerance and the propensity to physical activity. Mitochondrial respiration was significantly increased in cardiomyocytes isolated from treated db/db mice, as well as in diabetic cardiomyocytes treated with Arg in vitro. The improvement of cardiac mitochondrial function in db/db + Arg mice was associated with an increase in PGC-1-alpha levels, mitochondrial biogenesis, recycling, and antioxidant capacity. Arg treatment prevented the accumulation of circulating and cardiac miR-143 in db/db mice, which is an index of metabolic stress and activation of mitochondrial damage mechanisms. In conclusion, Arg supplementation is effective in preventing the development of DC, preserving diastolic function and exercise tolerance by improving mitochondrial fitness and homeostasis. Additionally, miR-143 could potentially be employed to monitor cardiac metabolic stress and the effects of Arg treatment in diabetes.

**Supplementary Information:**

The online version contains supplementary material available at 10.1186/s12933-024-02490-x.

## Introduction

Diabetic cardiomyopathy (DC) represents a cardiac metabolic dysfunction characterized by pathological left ventricular hypertrophy (LVH) independent of hypertension, coronary disease, and other cardiovascular conditions [1, 2]. DC is the leading cause of heart failure (HF) and mortality in diabetic patients [3]. In the first stage of DC, the symptoms are often inconspicuous, with patients presenting diastolic dysfunction and exercise intolerance often neglected [1]. In particular, the early metabolic and morphological (adaptive and maladaptive) remodeling of diabetic heart induces the development of HF with preserved ejection fraction (HFpEF), which can eventually evolve in classic, severe and irreversible HF. Hence, the diagnosis and treatment of DC in the early phase is extremely important to prevent irreversible metabolic remodeling, energetic collapse, and lethal HF evolution. However, drugs that specifically act on cardiac damage in diabetes are missing and most of diabetic patients inevitably develop HF. Moreover, the intricate cellular and molecular mechanisms that are involved, already in the early phases of DC, are still not fully elucidated.

The missing information on the molecular events that orchestrate metabolic stress response in diabetic heart makes difficult the identification of early biomarkers and the design of specific treatments. Data from recent studies conducted in different animal models indicate that mitochondrial dysfunction has a central role in DC pathogenesis, and that mechanisms involved in the control of mitochondrial function are early activated in prediabetic stage [4]. Mitochondrial abnormalities including focal damage and ultrastructural derangements, alongside with alterations in respiratory capacity, PGC-1alpha dependent mitochondrial biogenesis and antioxidant power [4, 5] have been reported in DC. In particular, diastolic dysfunction in the diabetic heart is strictly associated with mitochondrial alterations [5]. Moreover, miRNAs regulating mitochondrial function and metabolic stress response have been associated with the development of DC [6]. For example, miR-143 is implicated in metabolic remodeling and mitochondrial damage during stress conditions [7] and has been associated with major risk of metabolic syndrome and diabetes development [8].

In this scenario, an effective prevention and treatment of DC requires strategies that promote mitochondrial function, and that counteract the metabolic stress induced by gluco- and lipo-toxicity in diabetic heart.

Arg supplementation is known to improve mitochondrial function, dynamism, and energetics [9, 10]. Specifically, Arg acts on several metabolic pathways supporting mitochondrial and energetic homeostasis. The effects of Arg supplementation on the energetic capacity seem to be independent from age, previous diseases and lifestyle; indeed, beneficial action of Arg has been recorded in young sportive subjects, as well as in elderly populations, and in patients with angina pectoris [11]. Moreover, chronic Arg administration attenuated cardiac hypertrophy in spontaneously hypertensive rats [12], suggesting a potential beneficial action of Arg on the mechanisms underlying cardiac pathologic remodeling.

On these ground, we sought to evaluate the effects of Arg supplementation on DC onset and progression, determining its impact on mitochondrial homeostasis in diabetic heart during the early stage of DC. We employed db/db mice as model of Type 2 Diabetes, which are known to develop HFpEF [13], determining the effects of chronic Arg administration on LVH, diastolic dysfunction, and exercise intolerance. Finally, we evaluated the impact of Arg on cardiac mitochondrial homeostasis and energetics, as well as on miR-143 levels in diabetic mice.

**Fig. 1:**
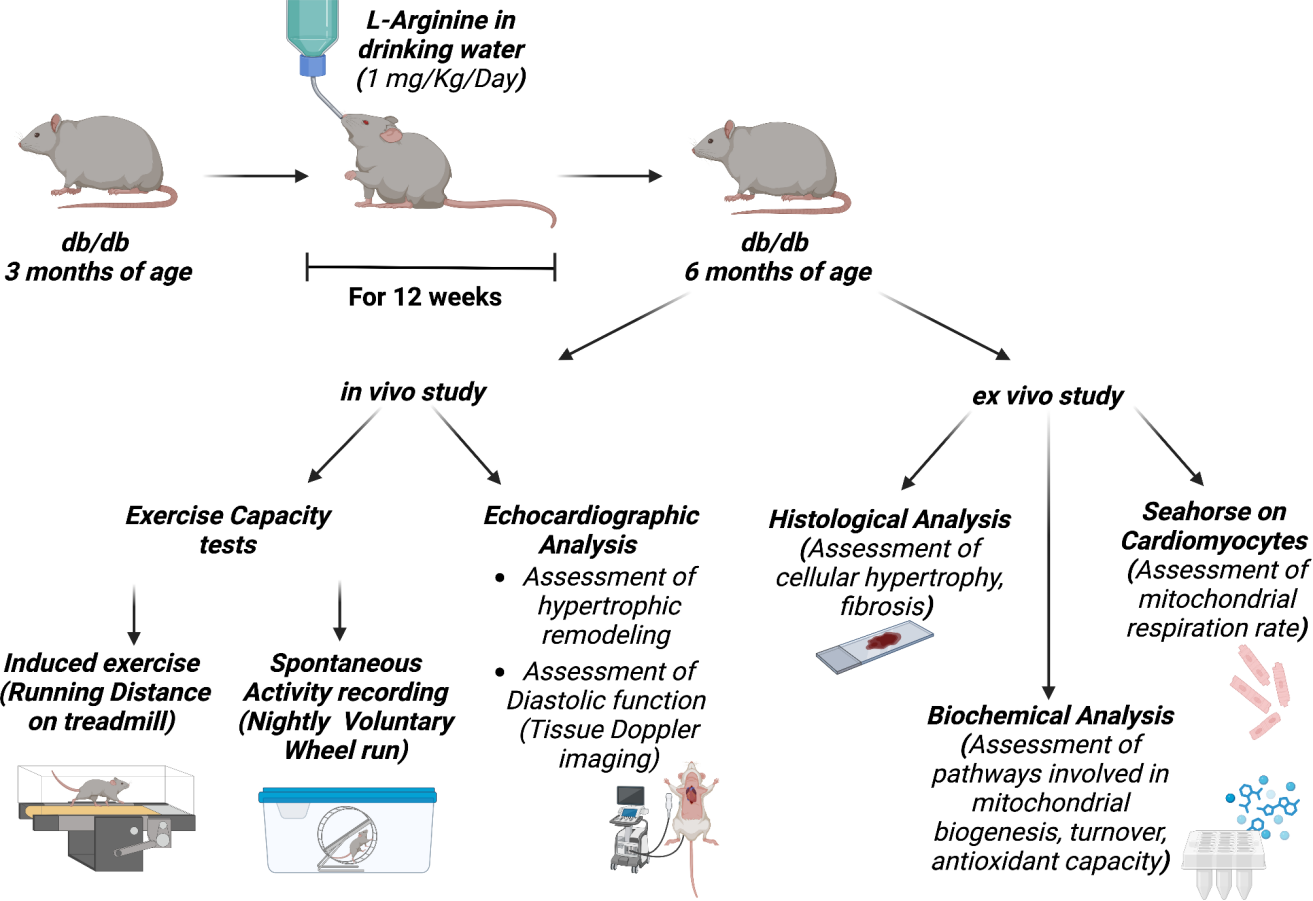
Study Protocol. Starting from 3 months of age db/db mice were treated with Arg in drinking water at the indicated dose. After 12 weeks of treatment, cardiac function and remodeling and exercise capacity were assessed in vivo. Rodents were sacrificed and ex vivo experiments performed: histological and biochemical analysis on cardiac tissue, as well as mitostress test on isolated cardiomyocytes by Seahorse.

The study protocol is depicted in Fig. 1.

## Results

### Arginine supplementation prevents cardiac hypertrophic remodeling and diastolic dysfunction in db/db mice

**Fig. 2:**
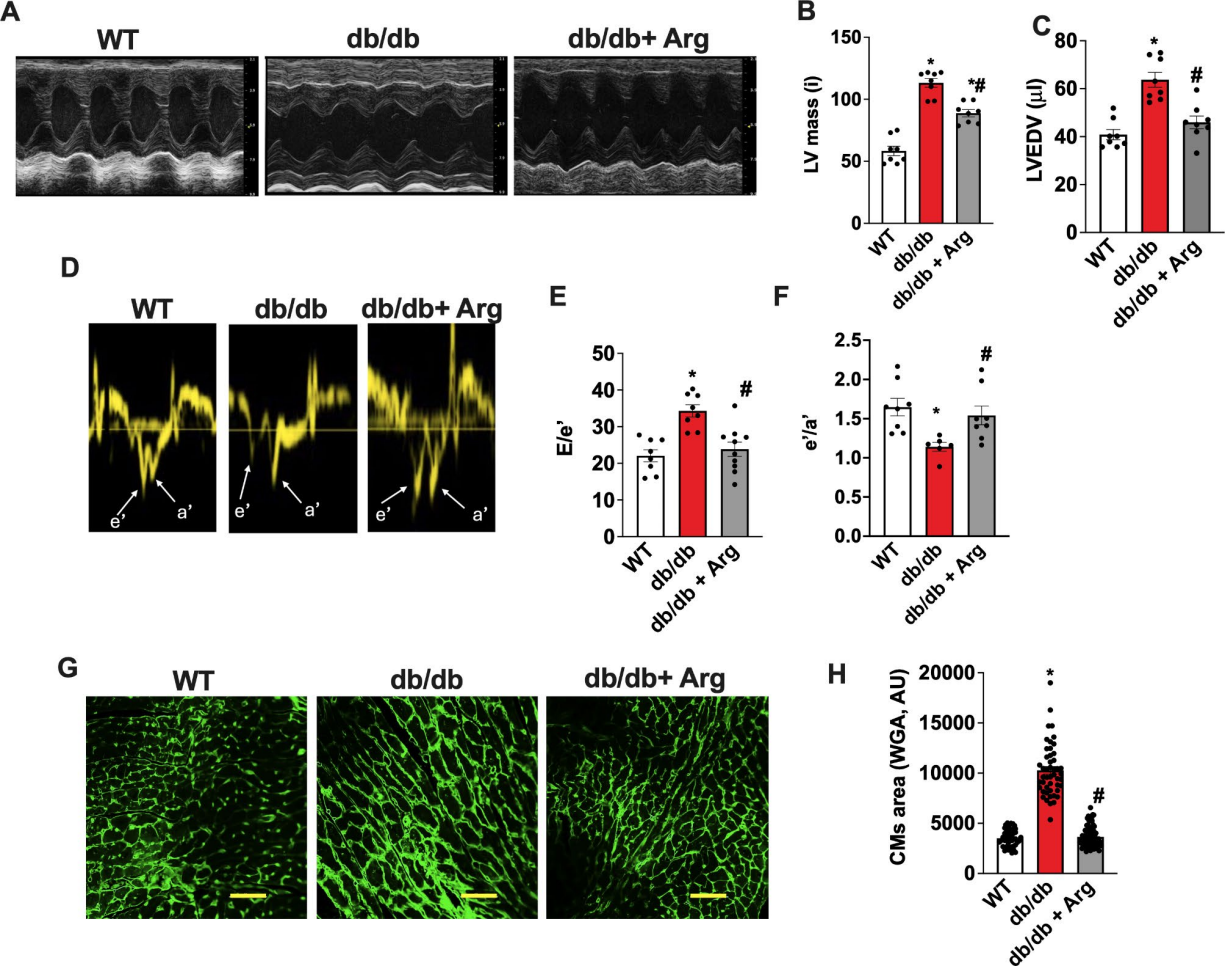
Echocardiographic analysis of wt, db/db, and Arg-treated db/db mice showing: M-mode representative images for short axis (**A**), Left Ventricular Mass automatically determined by echocardiography machine (LVmass (normalized by body weight) (**B**), Left Ventricular end Diastolic Volume (LVEDV) (**C**). Tissue doppler imaging was performed to evaluate diastolic function. Representative images of e’ and a’ waves (**D**). The automatically calculated e′/a′ (**E**) and E/e′ (**F**) ratio were displayed for each group. WGA staining was performed to determine cellular hypertrophy. Representative images for WGA staining on cardiac histologic sections (**G**), and cumulative quantification of cardiomyocytes cross sectional area (**H**). ANOVA followed by Bonferroni correction was used to assess the significative differences among groups. (WT, db/db, db/db + Arg *n* = 8) *p < 0.05 vs WT, # *p* < 0.05 vs db/db.

Previous reports indicate that 6-month-old db/db mice develop the main signs of diabetic cardiomyopathy, including left ventricular (LV) hypertrophy and diastolic dysfunction [13]. Accordingly, our echocardiographic analysis indicated a significant increase of cardiac mass and LV-end-diastolic volume (LVEDV), alongside with the reduction of e′/a′ and the increase of E/e′ ratio in db/db mice compared to age matched controls (Fig. 2A–F; Table 1). To evaluate the effects of Arg supplementation in preventing the development of diabetic cardiomyopathy, we supplemented 12-weeks-old db/db mice with Arg in drinking water, 1 mg/Kg/day for 3 months. The echocardiographic data indicated that LV mass and LVEDV were significantly lower in Arg treated db/db compared with untreated mice, indicating attenuation of LV remodeling (Fig. 2A-C). Additional echocardiographic parameters, including LV and Intra-ventricular septum diameters (LVEDD-LVESD, IVSd) also confirmed the therapeutic effects of Arg in preventing maladaptive heart remodeling in db/db mice (Table 1). Tissue Doppler imaging revealed a significant recovery of e′/a′ ratio in Arg treated db/db mice, alongside with a reduction of E/e′ ratio, hence suggesting that Arg treatment was able to preserve diastolic function (Fig. 2D–F). The effects of Arg in inhibiting cardiac hypertrophic remodeling was confirmed ex vivo by measuring heart weight / tibia length ratio (Table 1), as well as by determining cardiomyocytes cross-sectional area in isolated tissues by WGA staining (Fig. 2G–H). Consistently, Arg supplementation also prevented collagen deposition in db/db heart (Fig. S1A, B).

**Table 1:** Morphological and functional cardiac parameters

	HW/ Tl (g/mm)	LVEDD (mm)	LVESD (mm)	FS (%)	IVSd (mm)
WT	0.007058±0.000651	3,08 ± 0,1	1,22 ± 0,1	60,1 ± 2,9	0,95 ± 0,4
db/db	0.009306±0.000617	3,45 ± 0,2^*^	2,07 ± 0,2^*^	53,1 ± 6,6	0,74 ± 0,4*
db/db + Arg	0.007694±0.0002^!^	3,11 ± 0,09^!^	1,22 ± 0,08^!^	60,7 ± 4,7	0,94 ± 0,6!

*HW/tl* heart weight/ tibia length (g/mm), *LVEDD (mm)* left ventricular end-diastolic diameter, *LVESD (mm)* left ventricular end-systolic diameter, *FS (%)* fractional shortening, *IVSd (mm)* interventricular septum thickness at end-diastole.

The values are presented as average ± DS. *= p<0.05 vs WT,!= p<0.05 vs db/db.

## Arginine supplementation improves exercise tolerance in db/db mice

**Fig. 3:**
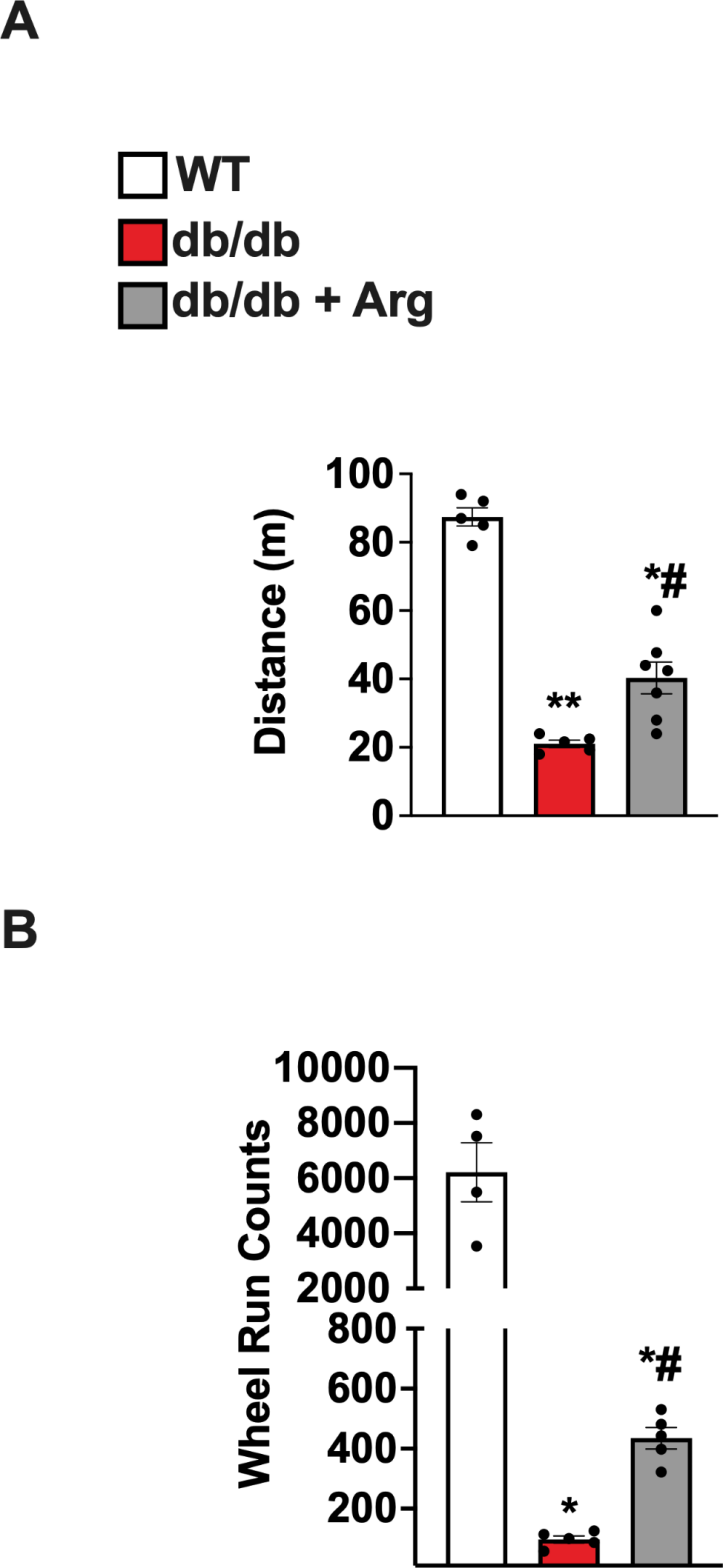
Exercise tolerance was assessed by induced running on treadmill, with increasing speed to exhaustion. The total distance covered by running was calculated for each mouse (WT *n* = 5, db/db *n* = 5, db/db + Arg *n* = 7) (**A**). The propensity to physical activity was determined by spontaneous overnight wheel run. The automatically calculated count of wheel runs is displayed (WT *n* = 4, db/db *n* = 5, db/db + Arg *n* = 5) (**B**). ANOVA followed by Bonferroni correction was used to assess the differences among groups. *p < 0.05 vs. WT, # *p* < 0.05 vs. db/db.

Severe exercise intolerance is a well-established hallmark of diastolic dysfunction and is a common feature of diabetic patients, with a strong predictive power for cardiovascular and all-cause mortality [14]. To evaluate the effects of Arg on exercise capacity, we assessed the tolerance to induced exercise as well as the propensity to spontaneous physical activity in WT, treated and untreated db/db mice. In end point (at the end of Arg treatment and after in vivo cardiac study), mice were placed on the treadmill with increasing speed to exhaustion. Maximum speed and running time were monitored, and the total distance was calculated based on the individual performance. Arg treated db/db mice exhibited a significant improvement in running capacity compared to untreated group (Fig. 3A). The propensity to spontaneous activity was assessed by measuring the overnight wheel-run. Accordingly, Arg treated db/db mice exhibited a marked improvement of the overnight running behavior (Fig. 3B). Interestingly, Arg treatment did not seem to affect the diabetic phenotype in terms of obesity, glycemia, and glucose intolerance (Fig. S2A–C). Overall, these data indicate that Arg exposure improves exercise tolerance in diabetic mice without affecting glucose homeostasis and insulin resistance.

## Arginine improves mitochondrial function and energetics in diabetic heart

**Fig. 4:**
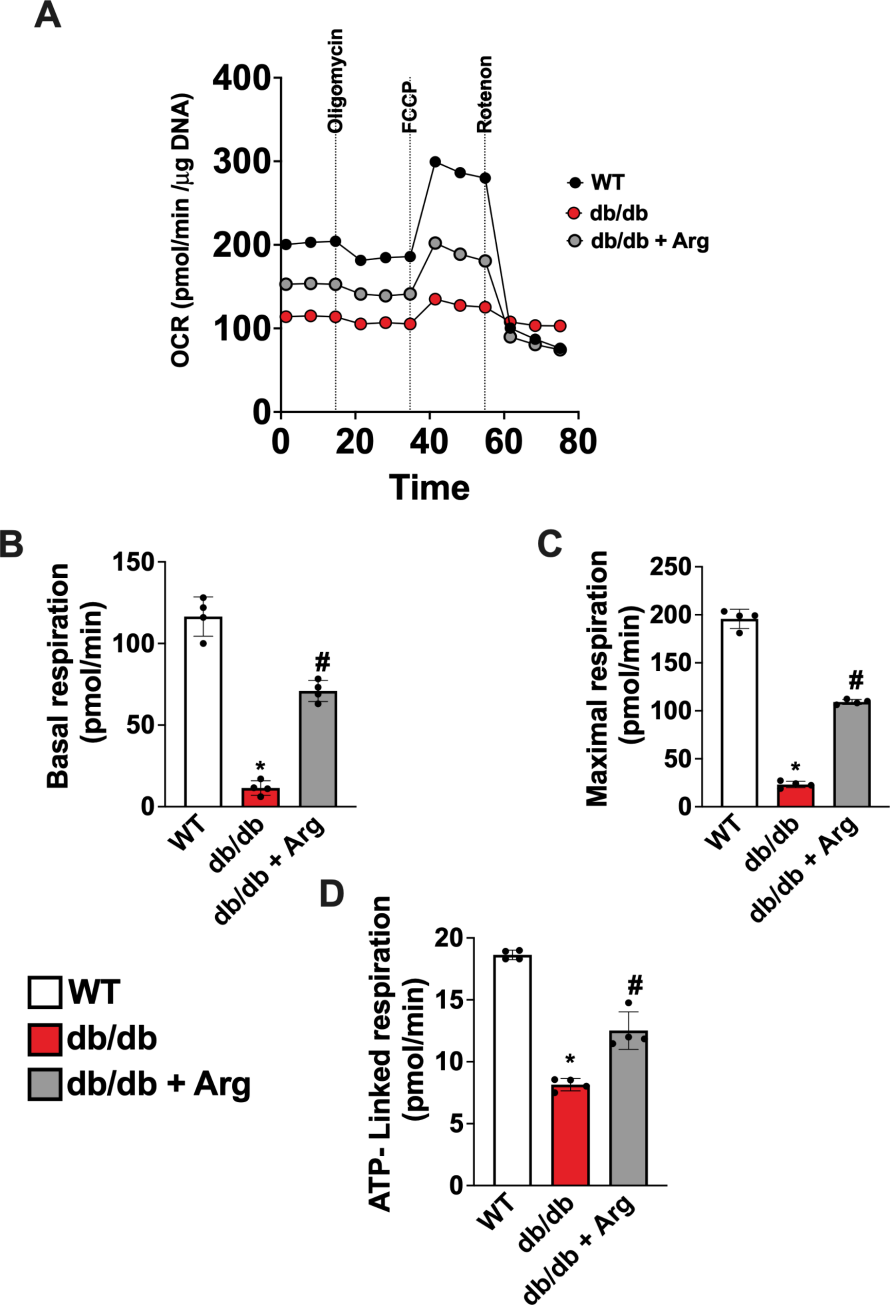
Adult Ventricular cardiomyocytes were isolated from WT, db/db, and fArg-treated db/db mice. The day after isolation, the Oxygen Consumption Rate was determined by Seahorse, in basal conditions and in response to Oligomycin, FCCP and Rotenone (**A**). Basal (**B**) and maximal respiration rate (**C**), alongside with ATP- coupled respiration (**D**) were determined. ANOVA followed by Bonferroni correction was used to assess the significative differences among groups (WT, db/db, ddb/db + Arg *n* = 4). *p < 0.05 vs. WT, # *p* < 0.05 vs. db/db.

Mitochondrial dysfunction is reported as a key molecular mechanism underling HFpEF, including the pathogenesis of diabetes-related HFpEF. To assess the mitochondrial effects of Arg in the diabetic heart, we isolated adult ventricular cardiomyocytes (CMs) from WT, Arg-treated, and untreated db/db mice and we evaluated mitochondrial function by measuring the oxygen consumption rate (OCR) during mitostress test at Seahorse. The OCR profile of db/db CMs was dramatically altered compared with WT cells, and the alterations were significantly attenuated in CMs isolated from Arg treated mice (Fig. 4A). Specifically, maximal and minimal respiration rates as well as ATP- production coupled respiration were significantly ameliorated in CMs from treated mice (Fig. 4B–D). Similar results were obtained when CMs, from untreated db/db, were exposed to Arg in vitro for 24 h, indicating a direct effect of Arg in improving mitochondrial function and energetics in cardiac cells, independent from a potential Arg systemic action, in vivo (Fig. S3 A–D).

Overall, our data showed a significant impact of Arg on cardiac energetics, a key target mechanism to solve the diastolic dysfunction and related phenotypes, including exercise intolerance [15, 16].

## Arginine induces PGC-1α upregulation supporting mitochondrial homeostasis in the diabetic heart

**Fig. 5:**
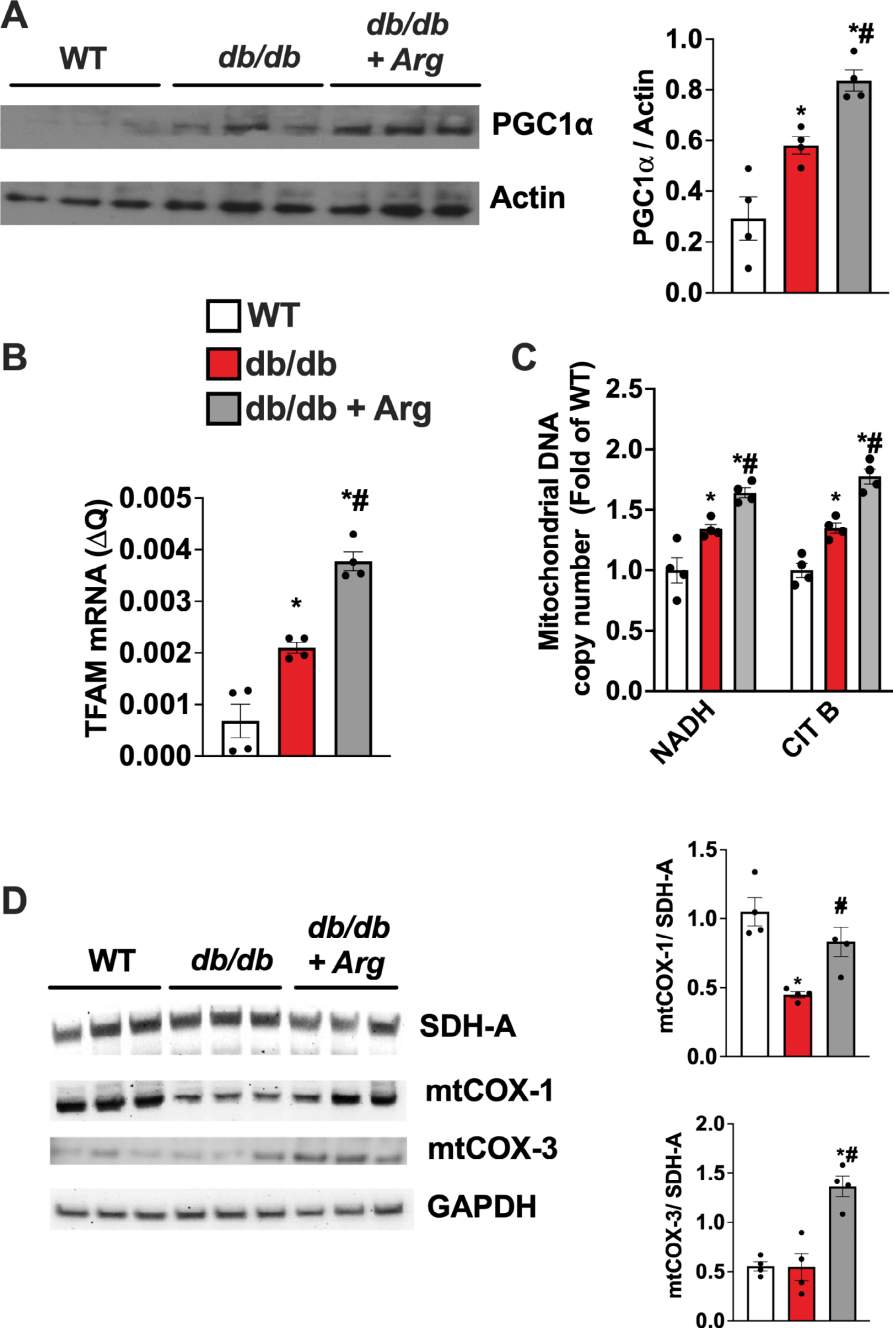
Western blot analysis of PGC1 alpha in cardiac lysate, and relative quantification using Actin as loading control (**A**). Real-time PCR for TfaM mRNA levels in cardiac extract (**B**). Mitochondrial biogenesis determined by assessing the copy number of mitochondrial genes NADH and Cytochrome B by Real-time PCR, using a nuclear gene (GAPDH) as control (**C**). Active expression of mitochondrial genes was determined by evaluating the levels of a mitochondrial proteins encoded by mitochondrial DNA, mtCOX-1 and mtCOX-3, compared with the levels of a mitochondrial protein encoded by nuclear genome SDH-A; GAPDH was used as loading control (**D**). The images are representative of 3 independent experiments (WT, db/db and db/db + Arg *n* = 4) ANOVA followed by Bonferroni correction was used to assess the significative differences among groups. *p < 0.05 vs. WT, # *p* < 0.05 vs. db/db.

The ability of Arg to support mitochondrial function has been linked to the induction of Peroxisome proliferator-activated receptor γ coactivator 1α (PGC-1α) [9, 17, 18]. Indeed, PGC-1α is the key player in regulating mitochondrial biogenesis and function, mitochondrial network, dynamism, mitophagy, and antioxidant defense [19]. In order to explore the mechanism by which Arg improves mitochondrial function in diabetic CMs, we assessed the expression levels of PGC-1α and the related pathways supporting the mitochondrial homeostasis. According to previous reports, PGC-1α levels were increased in diabetic hearts compared to controls, probably as an adaptive response to the ATP wasting and energetic collapse [20, 21]. In the hearts from Arg treated db/db mice, PGC-1α further increased, significantly (Fig. 5A). In line with these findings, the transcriptional levels of Tfam, a key PGC-1α target gene also increased (Fig. 5B), alongside with activation of mitochondrial biogenesis (Fig. 5C) and expression of mitochondrial genome encoded proteins like mtCOX-1 and mtCOX-3 (Fig. 5D).

**Fig. 6:**
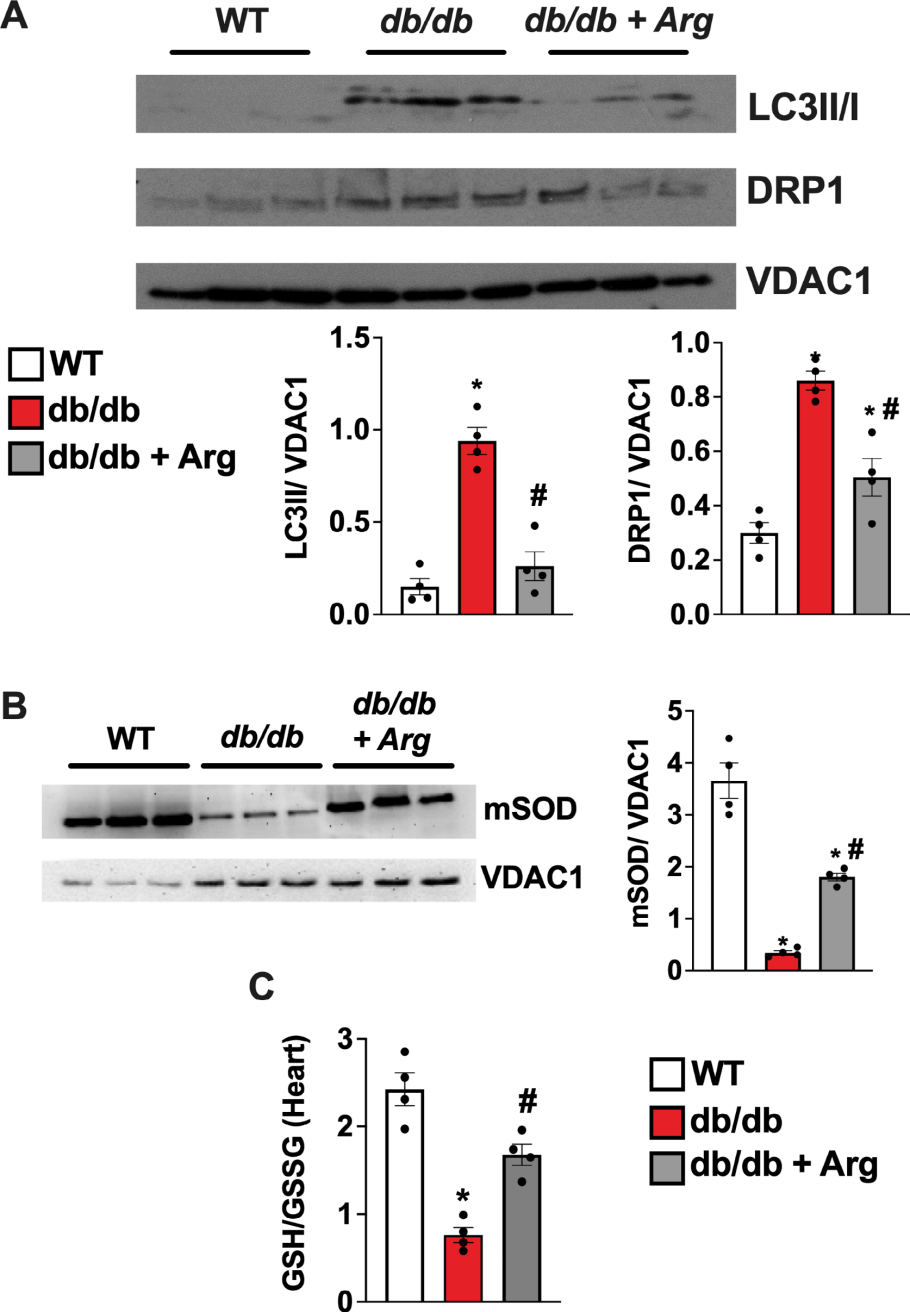
Mitochondria were isolated from cardiac tissue of wt, db/db and treated db/db mice and western blot analyses on mitochondrial extract was conducted to evaluate the levels of LC3II, DRP1 (**A**) and SOD (**B**). VDAC1 was used as loading control for mitochondrial extracts. To assess redox status GSH/ GSSG ratio was determined by immunoenzymatic based assay conducted on cardiac tissue from WT, db/db and Arg treated db/db mice (**C**). The images are representative of 3 independent experiments. (WT, db/db, db/db + Arg *n* = 4). ANOVA followed by Bonferroni correction was used to assess the significative differences among groups. *p < 0.05 vs. WT, # *p* < 0.05 vs. db/db.

Accordingly, mitochondria from treated diabetic mice displayed reduced accumulation of DRP1 and LC3 levels compared with mitochondria from untreated mice, confirming the improvement in mitochondrial dynamism and recycle (Fig. 6A). Moreover, mitochondrial superoxide dismutase (SOD) levels were restored in treated db/db hearts (Fig. 6B), alongside with the ratio of reduced vs. oxidized glutathione (GSH/GSSG) indicating an improvement of mitochondrial antioxidant capacity and a balanced redox status in Arg treated mice (Fig. 6C).

Skeletal muscle energetics is also a crucial determinant of exercise tolerance [22]. Hence, we also evaluated Arg potential effects on skeletal muscle energetics in db/db mice. Arg induced a significant increase of muscular ATP content in db/db mice (Fig. S4A). Moreover, in skeletal muscle too, Arg increased the levels of PGC1alfa, the master regulator of mitochondrial biology and homeostasis (Fig. S4B), without affecting myofibers transition in diabetic skeletal muscle (Fig. S4C–E).

This evidence suggest that the muscular effects of Arg, alongside the cardiac action can synergically contribute to the improvement of energetic and exercise capacity in treated mice.

## miR-143 is upregulated in DCM and Arginine supplementation reduces its level

The activation/inactivation status of molecular pathways involved in metabolic and mitochondrial dysregulation during cardiac adaptive/ maladaptive responses, is straightly dependent on the specific time point investigated [23]. The timing is also extremely important for guaranteeing the efficacy of therapeutic intervention using specific modulators of cellular metabolism and energetics. Therefore, early diagnostic biomarkers are essential to monitor the metabolic status and the energetic stress signaling, allowing to start therapeutic intervention before that maladaptive mechanisms become irreversible. MicroRNAs are being sought as biomarkers for the early identification of DC, and among them, miR-143 appears to be a good candidate. Indeed, in humans and in several mouse models the upregulation of miR-143 has been associated with higher metabolic risk and altered mitochondrial function [24]. Additionally, several targets of miR-143 have been identified, most of them linked to mitochondrial homeostasis and energetic metabolism including hexokinase and bcl2 [7, 25, 26].

**Fig. 7:**
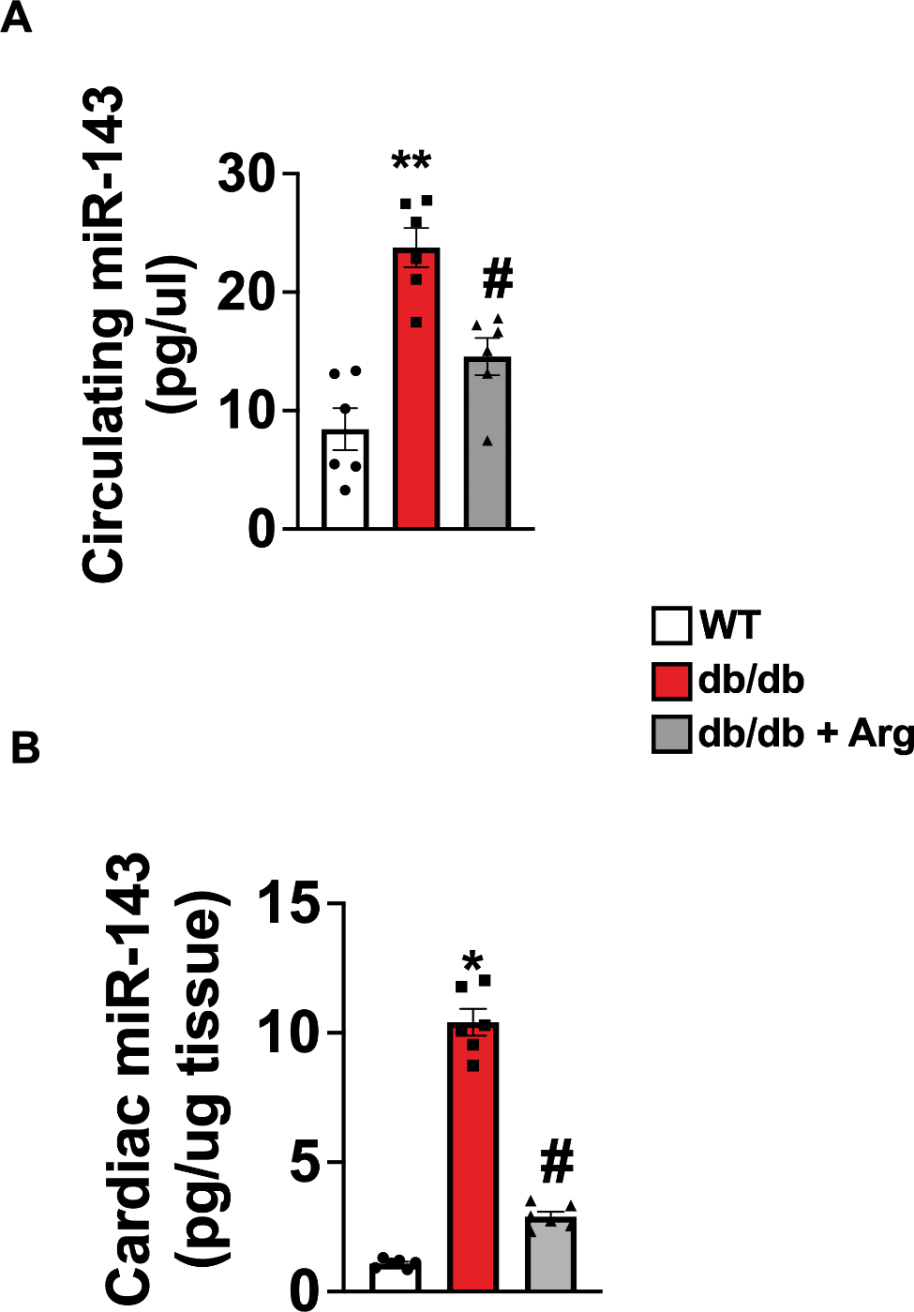
Measurement of microRNA-143 (miR-143) levels in plasma (**A**) and in cardiac tissue (**B**) isolated from wt, db/db, and treated db/db mice. (WT, db/db, db/db + Arg *n* = 6) ANOVA followed by Bonferroni correction was used to assess the significative differences among groups. *p < 0.05 vs. WT, # *p* < 0.05 vs. db/db.

In our setting, the metabolic stress and specifically the cardiac mitochondrial dysfunction occurred alongside with a significant increase in circulating and cardiac levels of miR-143; strikingly, Arg treatment reduced miR-143 levels in both districts (Fig. 7).

Taken together, these data confirm the efficacy of Arg treatment in reducing the cardiac metabolic stress in diabetes and suggest that miR-143 could be potentially employed as biomarker to decide for Arg supplementation and to monitor its efficacy on metabolic stress in diabetes.

## Discussion

In the current report, we evaluated the impact of Arg supplementation on cardiac mitochondrial/ energetic dysregulation and on cardiac maladaptive remodeling and dysfunction occurring in diabetes.

Our data demonstrated that Arg supplementation could be an effective strategy to support mitochondrial function during the metabolic stress response of diabetic hearts, thus preventing the development of LVH and dysfunction. In this study we tested the effects of Arg supplementation on the early signs of diabetic cardiomyopathy which includes diastolic dysfunction and exercise intolerance. Arg exposure preserved the diastolic function in db/db mice, mirrored by a conserved cardiac geometry. Moreover, Arg supplementation improved the exercise tolerance of diabetic mice, both, endurance activity as well as spontaneous propensity to exercise. Exercise intolerance is a key hallmark of HFpEF, including of diabetes-induced HFpEF [27–30]. Also, exercise intolerance is a strong predictor for cardiovascular and all-cause mortality in diabetic patients [31, 32], and under this light, the positive impact of Arg on exercise performance is of great resonance. Specifically, the improvement of exercise capacity could be linked to both Arg dependent effects on cardiac as well as on skeletal muscle determinants of exercise tolerance. Indeed, skeletal muscle condition also plays a central role in determining exercise capacity of HF patients [33], and our data show that Arg was able to improve energetics and PGC1α levels also in this district.

Although there are therapeutic strategies available for DC, the treatment is mainly focused on controlling blood glucose and blood lipids, and there is a lack of effective drugs specifically targeting myocardial injury. The pathogenetic mechanisms of DC are intricate and are still not fully understood [28]. Chronic hyperglycemia exerts toxic effects on the myocardium during the progression of DC through direct and indirect pathways activation, inducing cardiac remodeling, diastolic and systolic dysfunction, and eventually severe heart failure [1]. Well-established mechanisms include myocardial energy depletion due to impaired ATP synthesis and mitochondrial uncoupling, as well as increased mitochondrial oxidative stress [34–36].

The key role of mitochondrial dysfunction in the pathogenesis of DC represents was the rationale to test the therapeutic effects of Arg on diabetic heart. Indeed, the ability of Arg to support mitochondrial function and energy generation has been extensively reported [9, 11]. In our setting, the chronic administration of Arg can preserve the coupling efficacy of oxidative phosphorylation, improving the energetics of diabetic cardiomyocytes. Most likely, the central node of Arg action is the activation of the PGC1 alpha signaling pathway able to improve the efficacy of electron transport chain, as well as to drive a sustained mitochondrial regeneration and antioxidant capacity [37, 38]. Indeed, an efficient mitochondrial biogenesis is extremely important for diabetic mitochondria, which are exposed to glucotoxicity and lipotoxicity, as well as to extreme workload induced by the obligated fatty acid oxidation in insulin-resistant cells [39, 40]. In line with this hypothesis, we also observed an increased expression of DRP1 and LC3 on the diabetic mitochondrial surface as an index of accumulation of damaged and depolarized mitochondria [41]. Arg was able to reduce this phenomenon, thus promoting mitochondrial recycling and turnover. In the literature, there are several indications about the beneficial effects of Arg in diabetes especially in the early stages of the disease, to prevent or at least slow down the organ damage [42]. The low serum Arg levels and its correlation with foot ulcers in diabetic patients, further support the rationale of using Arg supplementation [43]. However, for the most of the reports, Arg supplementation is considered a strategy to counteract the endothelial dysfunction in diabetes by increasing NO bioavailability [44–46]. To the best of our knowledge, no studies have been specifically focused on Arg potential effects on DC and related metabolic and energetic alterations. In this regard, we do not exclude that the therapeutic effects of Arg on cardiac and physical performance of diabetic mice was in part determined by an improvement of vascular function and hemodynamics, as well as to its direct action on skeletal muscle energetics. However, our data showing the amelioration of mitochondrial function in db/db cardiomyocytes in response to Arg treatment in vitro, demonstrate a specific and direct effect of Arg on mitochondrial homeostasis and energetics in diabetic cardiomyocytes.

Given the neglected initial symptoms, the intricate cellular and molecular mechanisms, and the lack of available drugs, it is necessary to explore early diagnostic biomarkers for diabetes complications, especially for DC. MicroRNAs are being sought as biomarkers for the early identification of type 2 diabetes [8, 47]. It has been established that about sixteen microRNAs met the criteria to be selected as biomarkers, including the miR-143 [8]. Specifically, miR-143 has a significant role in regulating mitochondrial function and cellular metabolism under stress conditions [26]. Interestingly, the miR-143 promoter has binding sites for PPARγ [25], suggesting the involvement of this miRNA in PGC-1-alpha signaling pathway. Among the target genes regulated by miR-143, PKCε, hexokinase and bcl-2 have been confirmed, and their downregulation by miR-143 has been associated with mitochondrial membrane potential dissipation, and mitochondrial-dependent apoptosis [7, 48, 49]. Accordingly, the upregulation of miR-143 indicates a major metabolic risk in patients with metabolic syndrome [24].

Our data show a marked upregulation of circulating miR-143 in db/db mice, as well as of its cardiac levels, evidencing the status of metabolic stress and the activation of mitochondrial damage mechanisms in diabetic cells. Arg was able to significantly reduce miR-143 levels, indicating its ability in counteract cardiac metabolic stress. Hence, our study suggests the potential use of miR-143 for the early detection of metabolic stress in diabetic cardiomyopathy, as well as for monitoring the effects of therapeutics (including Arg).

## Materials and methods

### Animals

Heterozygous mice for leptine receptor knockout (Lepr^+/-^) were breaded to obtain homozygous Lepr^+/+^ mice (db/db). Negative littermates were used as control wild type mice (WT). Genotyping was performed using standard protocols. db/db and WT mice were kept in a controlled environment, with 12:12 h light/dark period; 23 °C; 55–60% humidity; and free access to water and chow. In vivo experiments were approved and conducted following the Guide for the Care and Use of Laboratory Animals published by the US National Institutes of Health (NIH Publication No. 85 − 23, revised 1985) and approved by the Italian Ministry of Health, authorization number 724/2019-PR. Arg was administered in drinking water (1 mg/Kg/day), starting from 3 months of age for 12 weeks.

### Echocardiographic study

Transthoracic echocardiography was performed in end point using a dedicated small-animal high-resolution imaging system Vevo 2100 high-resolution imaging system (Visual-Sonics, Toronto, ON, Canada).

The procedure was performed as previously described [50, 51]. Briefly, the mice were anesthetized by isoflurane (4%) inhalation and maintained by mask ventilation (isoflurane 2%). All measurements were averaged on at least 5 consecutive cardiac cycles and analyzed by 2 experienced investigators blinded to treatment. The assessment of diastolic function was determined by doppler echocardiography and tissue doppler imaging. Specifically, images from the apical four-chamber view were acquired to assess LV filling and diastolic function. Transmitral LV inflow velocities were measured by pulsed-wave Doppler. Peak early E wave (E) and late A wave (A) filling velocities and the E-to-A ratio (E/A) were measured. TDI was obtained by placing a 1.0-mm sample volume at the medial annulus of the mitral valve. Analysis was performed for the early (e′) and late (a′) diastolic velocity. The mitral inflow E velocity-to-tissue Doppler e′ wave velocity ratio (E/e′) and tissue Doppler early e′ velocity-to-tissue Doppler late a′ velocity ratio were calculated as indexes of diastolic function [13]. The echocardiographic offline analysis was performed by a sonographer blinded to the study groups.

### Treadmill

Before the exercise training, 1 week of adaptation was performed. After the adaption period, the training protocol consists of running cycles on a treadmill (Columbus Instruments, Ohio) from a minimum speed which then increases every 2 min, with the incline of the machine being gradually raised from (5 to 15°) during exercise periods [52]. Mice are enforced to run (electrical solicitation) till exhaustion and the covered time and distance were monitored for each mouse.

### Spontaneous activity recording

Mice were allowed to run freely on the plastic wheel placed inside a standard mouse cage. After 1 day of adaption, the number of rotations was automatically recorded by the cage wheel running system provided by Columbus Instruments (Columbus, OH). The software acquired data on the total number of wheel revolutions performed every hour for a 16-hour overnight period. Following data collection, mice were returned to cages without a running wheel for at least 3 days before ex vivo experiments.

### Mitochondrial respiration (oxygen consumption rate)

Mitochondrial respiration was assessed as previously described [53, 54], on isolated cardiomyocytes using Seahorse Analyzer (XFe96 Agilent Technologies, Santa Clara, CA, USA); mouse cardiomyocytes, from treated and untreated db/db and WT mice were plated in Seahorse 96-well plate 24 h before the assay at a density of 2 × 10^4^ cells/well. For the assay, the XF medium (non- buffered DMEM medium, containing 10 mM glucose, 2 mM L‐glutamine, and 1 mM sodium pyruvate) was used and the OCR was measured under basal conditions and in response to different chemical stimulant and inhibitor compounds. In particular, 2.5 µM oligomycin (ATP synthase inhibitor), 1 µM carbonylcyanide‐4‐(trifluoromethoxy) phenylhydrazone (FCCP- mitochondrial uncoupling), and 0.5 µM rotenone (Complex 1 inhibitor) were used for the assay.

OCR rates were measured 3 times, both in basal conditions and after the injection of each compound and, based on the average of these measures, basal respiration, maximal respiration, and ATP linked respiration were calculated as previously described [55]. After each assay, cells were collected to quantify DNA using QuantiFluor dsDNA System (Promega, Madison, WI) in order to normalize.

### Western blot analysis

An equal amount of protein from each sample (30 µg) was used for immunoblot analysis. Briefly, whole extract or membrane proteins were separated by 4–12% SDS/PAGE gel and transferred to an Immobilon-P nitrocellulose filter (Millipore); the membranes were blocked in Tris-buffered saline containing 0.002 g/l Tween 20 (TBST) and 0.05 g/l non-fat dry milk. After blocking, the.

membranes were washed three times with TBST and then incubated overnight at 4 °C in 5% BSA.

TBST with primary antibodies specific for LC3II/I, DRP1, mSOD, PGC1alpha, mtCOX1-3 (Cell Signaling), Tubulin (Sigma) and VDAC (SantaCruz Biotechnologies).

Secondary peroxidase-conjugated antibodies (ImmunoReagents) were used to visualize the antigen-antibody complexes on a nitrocellulose filter by chemiluminescence.

A standard chemiluminescence reaction kit (Pierce) was used for autoradiography on film.

### Real-time PCR

Gene expression levels were determined by real-time reverse transcription-polymerase chain reaction (RT-qPCR) as previously described and validated [50, 54, 56, 57].

Briefly, RNA extraction was performed from 50 mg of cardiac tissue by TRIzol Reagent (Invitrogen, Carsbad, CA, USA). The amount of isolated RNA was dissolved in 50 µL of RNAse free water and the concentration was determined by a micro-Volume spectrophotometer (MaestroGen, Carson City, NV). 2 µg of RNA for each sample was retro-transcripted by a One-Step RT-PCR kit (Vilo, Invitrogen). The RT-qPCR was then performed with a StepOne System-Applied Byosistem (ThermoFisher Scientific) using SyberGreen as identification method. In each amplification tube, a total volume reaction of 20 µL was composed by: 20 ng of synthetized cDNA (in a volume of 2 µL), 10 µL of BrightGreen 2X qPCR MasterMix-ROX (Applied Biological Material - abm, Richmond, BC, Canada), and 2 µL of forward and reverse 500 nM stock primers. Each tube was prepared in triplicates, and the RT-qPCR assay for 18S and TFaM were performed (MtTFA rev 5’CTTCAGCCATCTGCTCTTCC3’, MtTFA for 5’CAAAAAGACCTCGTTCAGCA3’). RT-qPCR assay was also performed to assessed the transcription levels of Myosin heavy chains isoforms (MHC1, MHC2a, MHC2b), using the primer sequences previously described [58]. Each RT-qPCR cycle consisted of heating at 95 °C for 15 s, 60 °C for 30 s for annealing, and 72 °C for 1 min for the extension. At the end of the reaction, melting curve analysis was performed to evaluate the specificity of the amplification reaction for each primer pair. The gene expression levels for each target gene were determined using the comparative Ct method normalized for 18 S [59].

### Mitochondrial isolation from cardiac tissue

Cardiac tissue was disrupted by Dounce homogenizer in isolation buffer (IB pH7.4 200 mM sucrose, 1 mM EGTA-Tris, and 10 mM Tris-MOPS), as described [50, 60, 61]. The homogenate was spun at 800×g for 10 min; the supernatant was recovered and further centrifuged for 10 min at 8000×g and the resulting pellet, mitochondrial fraction, was collected while the supernatant was recovered as the cytosolic fraction.

The mitochondrial fraction was further purified by centrifuging twice at 8000×g for 10 min. The obtained pellet was clarified by centrifugation at 95,000×g for 30 min on a 30% Percoll gradient in IB. The obtained mitochondrial layer was washed free from Percoll and resuspended in IB. Protein concentration was determined by the bicinchoninate assay (Pierce). DRP1, LC3II- I levels in mitochondrial fraction were determined by western blot analysis using a specific antibody as described above. VDAC levels were used as a loading control.

### Mitochondrial biogenesis

We assessed mitochondrial biogenesis as we reported [50, 60, 61]). Briefly, cell DNA was isolated using commercially available reagents (DNAzol-Invitrogen). Real-time quantitative polymerase chain reaction (RT-PCR) was performed on DNA to amplify two mitochondrial (cytochrome b, NADHd), and one nuclear gene. All values obtained were normalized to the values obtained for housekeeping gene (18s). The reaction was visualized by Sybr Green Analysis (Applied Biosystem) on a StepOne instrument (Applied Biosystem). 

### Determination of GSH and GSSG levels

The ratio of Total Glutathione (T-GSH)/Oxidized Glutathione (GSSG) was determined by a commercial available Colorimetric Assay Kit (Elabscience) and used as index of antioxidant cellular capacity. Specifically, GSH/ GSSG was determined on cardiac tissue isolated from WT, db/db and db/db + Arg mice following manufacture instructions, as previously described [62]. At the end of the colorimetric reaction, the OD values at 412 nm were determined by Tecan Infinite200pro plate reader.

### Skeletal muscle dissection and preparation

Femoral quadriceps muscle was isolated as previously described [63], and immediately frozen in liquid nitrogen. 20 mg of frozen tissue were homogenized with a Polytron (Brinkman Instruments, Riverview, FL, USA) in ice-cold RIPA/SDS buffer (50 × 10^–3^ mol/L Tris-HCl (pH 7.5), 150 × 10^–3^ mol/L NaCl, 0.01 g/L NP-40, 0.0025 g/L deoxycholate, 2 × 10^–3^ mol/L Na_3_VO_4_, 0.2 g/L sodium, 2 × 10^–3^ mol/L EDTA, 2 × 10^–3^ mol/L PMSF), for whole protein extraction and western blot analysis. Alternatively, the collected tissue was lysated in TriZol for RNA isolation and RT-qPCR, or to assess ATP content using a commercially available kit (abcam).

### Blood collection and circulating miRs determination

Peripheral blood was collected from mice and plasma was obtained by centrifugation as previously reported. We extracted microRNAs using the using the miRNeasy Serum/Plasma kit (Qiagen, Hilden, Germany) according to the manufacturer’s protocol. The quality of miR was determined using Agilent Small RNA Kit [29, 30]. A custom panel of mitomiRs was quantified as we previously described [64].

### Cardiomyocytes isolation

Adult murine ventricular myocytes (AMVMs) were isolated by a standard digestion procedure using the Langendorff system, as previously described. [50, 60, 65].

### Statistical analysis

All values are presented as mean ± SEM. ANOVA was performed to compare the different parameters among the different groups (WT, db/db, db/db + Arg). Bonferroni post hoc testing was performed as appropriate. A significance level of *p* < 0.05 was assumed for all statistical evaluations. Statistics were computed with GraphPad Prism software (Dotmatics, Boston, MA).

## Electronic Supplementary Material

Below is the link to the electronic supplementary material.

Supplementary Material 1

## Data Availability

The data that support the findings of this study are available from the corresponding authors upon reasonable request.

## Declarations

### Licenses

Figure 1 was created in BioRender. License was granted to Iaccarino, G. (2024- BioRender.com/c38b746).
