# Supplementary material for: L-Arginine supplementation as mitochondrial therapy in diabetic cardiomyopathy
Source: Cardiovasc Diabetol. 2024 Dec 20;23:450. doi: 10.1186/s12933-024-02490-x (PMC11662564; doi:10.1186/s12933-024-02490-x)
Supplement: Supplementary file 1 — Supplementary Material 1 [file 12933_2024_2490_MOESM1_ESM.pdf]

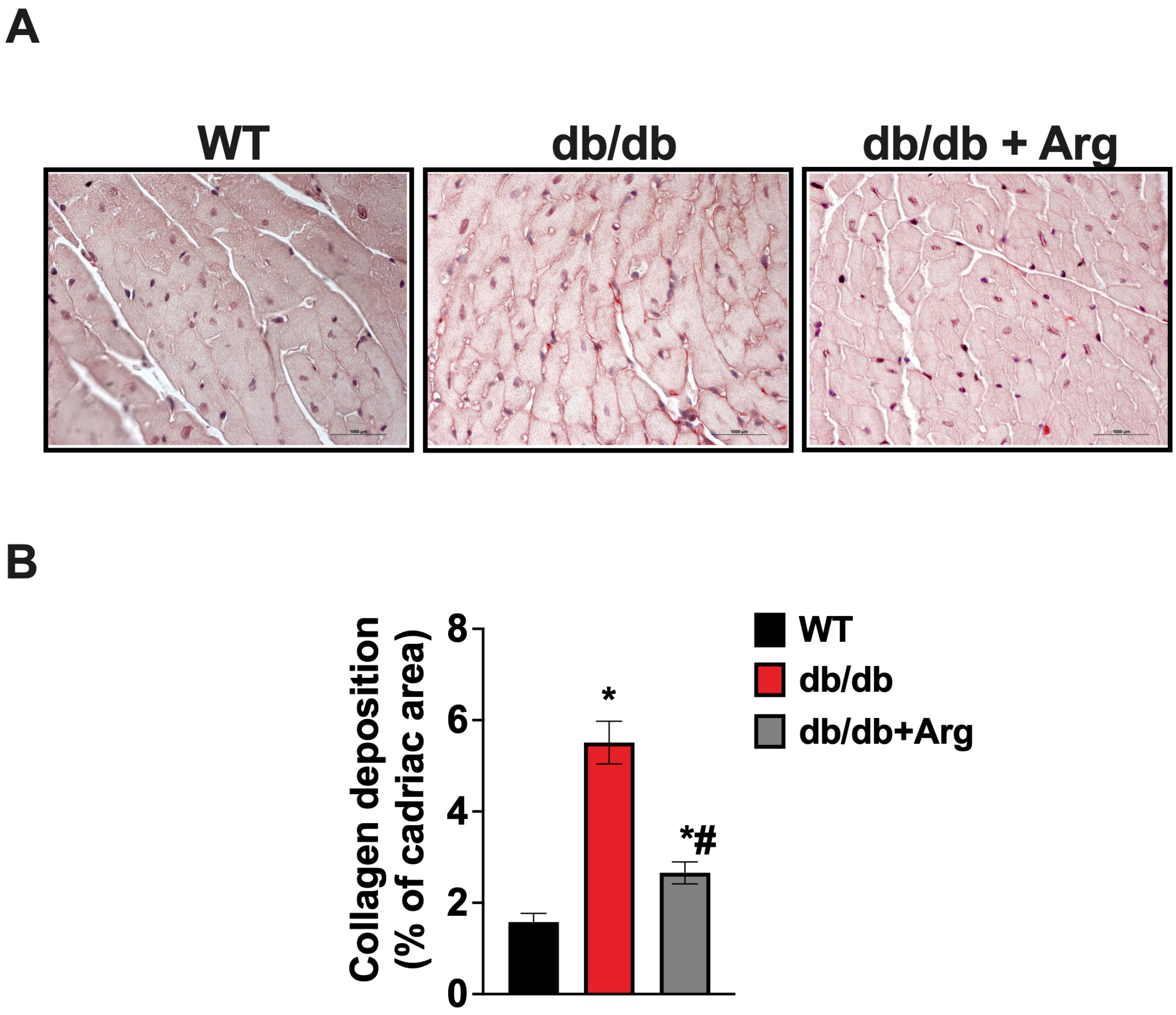

Figure S1.

Representative Picrosirius Red staining for collagen deposition in the left ventricle; dimensional bar: 1000 µm. (**A**). Quantitative data for total collagen deposition (**B**). *p<0.05 vs WT, # p<0.05 vs db/db.

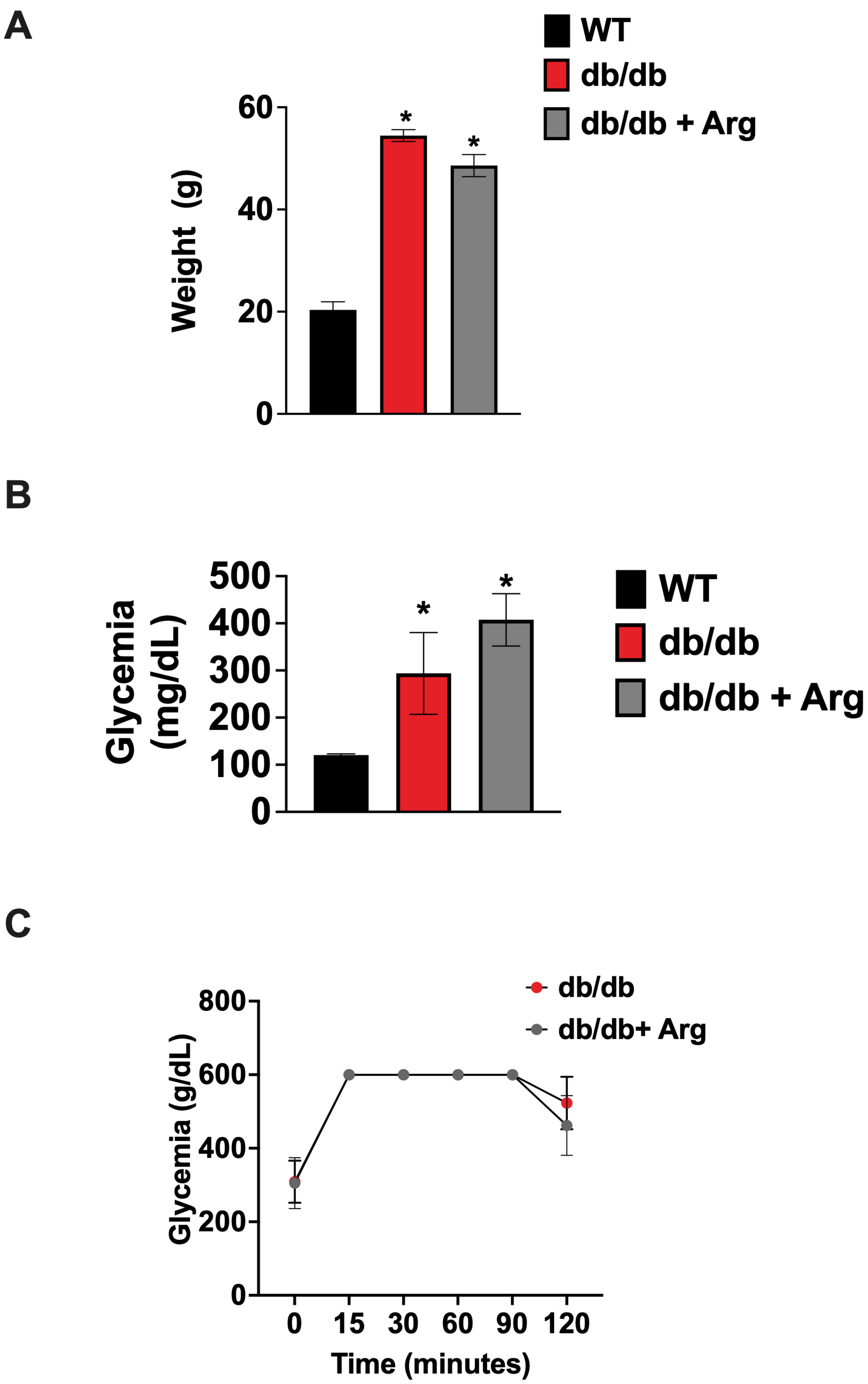

Figure S2.

Evaluation of diabetes-related phenotypes at end-point. Body weight (**A**), glycemia (**B**), glucose tolerance test at the indicated time points (**C**). ANOVA followed by Bonferroni correction was used to assess the significative differences among groups (WT, db/db, db/db + Arg; n=4). *$p<0.05$ vs WT, # $p<0.05$ vs db/db.

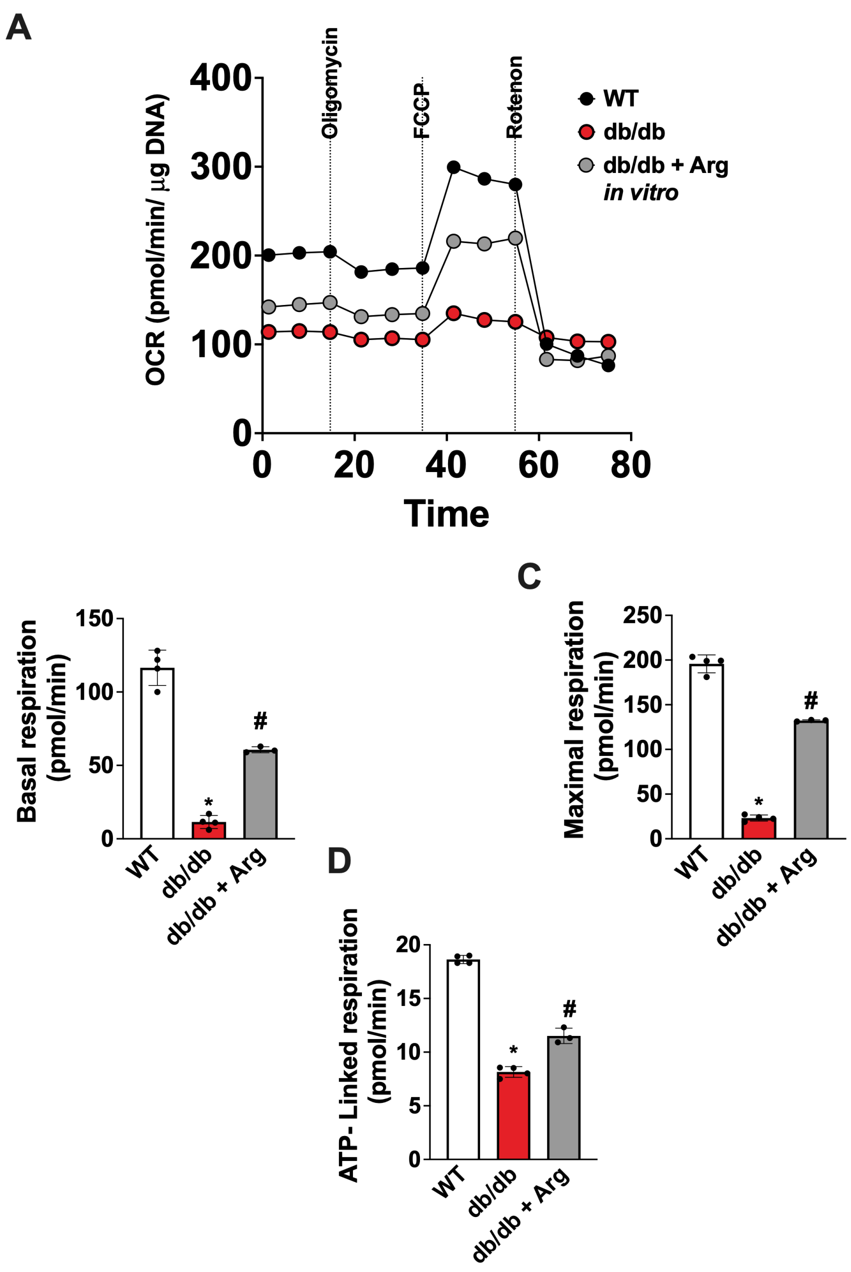

Figure S3

Adult left ventricular myocytes were isolated from WT and db/db mice. Immediately after attachment (1 hour), Arg was directly added to cell medium (50 µM) when indicated (Arg *in vitro*). The day after, oxygen consumption rate (OCR) was determined by Seahorse, in basal conditions and in response to Oligomycin, FCCP, and Rotenone (**A**). Basal (**B**) and maximal respiration rate (**C**), alongside ATP-coupled respiration (**D**) were determined. ANOVA followed by Bonferroni correction was used to assess the significative differences among groups (WT, db/db n=4, db/db +Arg *in vitro* n=3). *$p<0.05$ vs WT, # $p<0.05$ vs db/db.

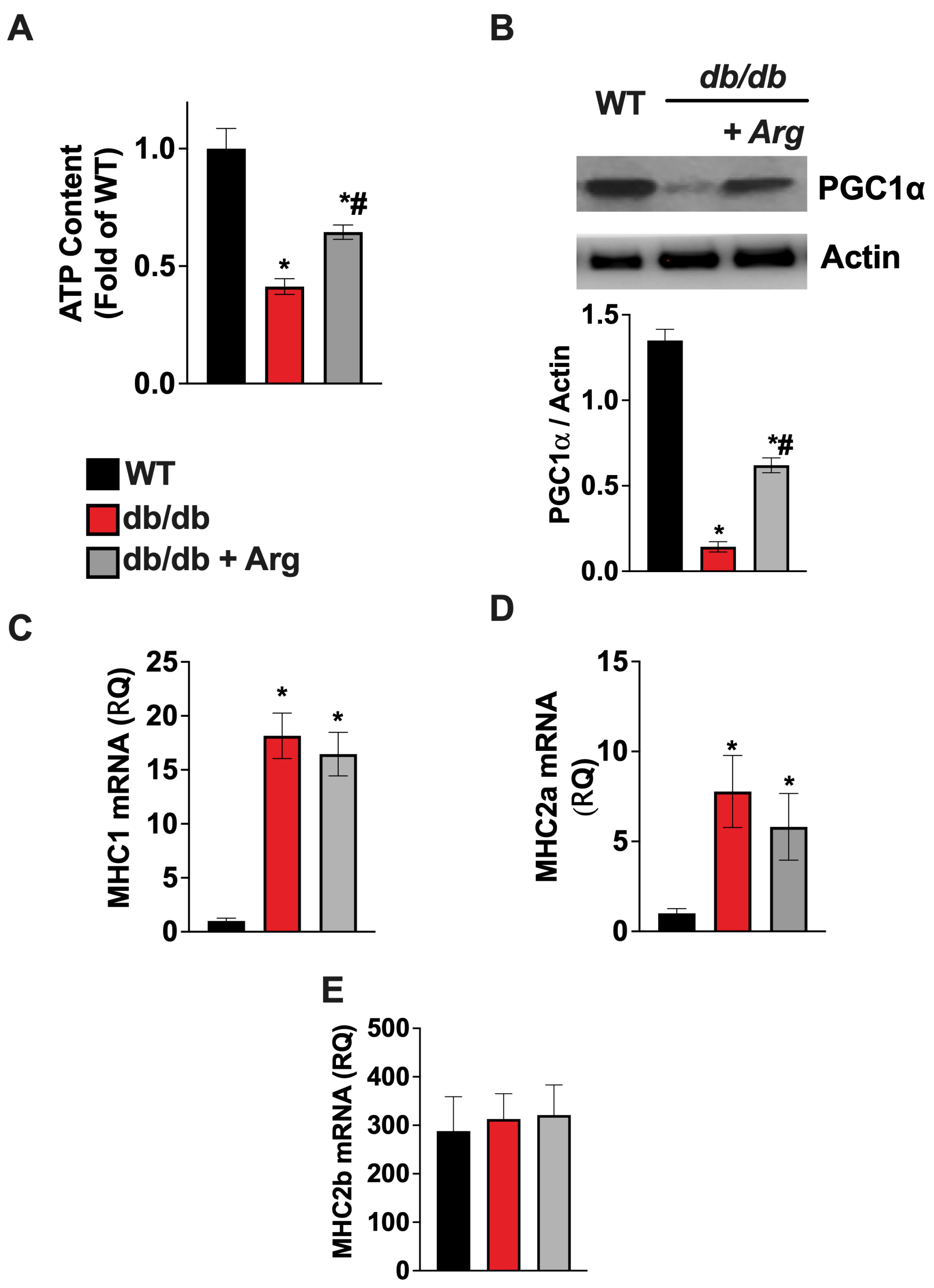

Figure S4

ATP content was determined in skeletal muscle lysate and expressed as fold of control (**A**). Western blot analysis on whole muscle lysate for PGC1alpha detection. GAPDH was used as loading control (**B**). In order to assess muscle fibres transition, Real-time PCR for mRNAs of Myosin isoforms MHC1, MHC2a, MHC2b, (**C-E**) was conducted. The images are representative of at least 3 independent experiments. ANOVA followed by Bonferroni correction was used to assess the significative differences among groups (WT, db/db, db/db +Arg n=4). *$p<0.05$ vs WT, # $p<0.05$ vs db/db.
